# Stability-Indicating High-Performance Liquid Chromatographic Determination of Apixaban in the Presence of Degradation Products

**DOI:** 10.3797/scipharm.1403-25

**Published:** 2014-05-22

**Authors:** Swarup Suresh Prabhune, Rajendra Shankar Jaguste, Prakash Laxman Kondalkar, Nitin Sharadchandra Pradhan

**Affiliations:** Analytical development laboratory, Wanbury R&D center, Plot No.EL-16, Mahape, New Mumbai 400710, India.

**Keywords:** HPLC, Apixaban, Reversed phase, Stability-indicating

## Abstract

A simple, robust, and stability-indicating reversed-phase high-performance liquid chromatographic (HPLC) method for the analysis of apixaban and its related substances has been successfully developed. Chromatography was performed on a 250 mm × 4.6 mm, 5 μm C_18_ column with a gradient mixture of a phosphate buffer–methanol 60:40 (v/v) at 1.0 mL min^-1^. Ultraviolet detection of apixaban was at 220 nm. The method was validated for linearity, precision, repeatability, sensitivity, and selectivity. Selectivity was validated by subjecting apixaban solution to photolytic, acidic, basic, oxidative, and thermal degradation. The peaks from the degradation products did not interfere with that from apixaban. The method was used to quantify the related substances in apixaban in the bulk drug and can be used for routine quality control purposes.

## Introduction

**Fig. 1.**
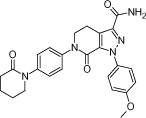
The structure of apixaban

Apixaban, 1-(4-methoxyphenyl)-7-oxo-6-[4-(2-oxopiperidin-1-yl)phenyl]-4,5,6,7-tetrahydro-1*H*-pyrazolo[3,4-*c*]pyridine-3-carboxamide [1], is indicated to reduce the risk of stroke and systemic embolism in patients with nonvalvular atrial fibrillation. The molecular structure is shown in Fig. 1.

The FDA approved apixaban in December 2012 with an indication of reducing the risk of stroke and dangerous blood clots (systemic embolism) in patients with atrial fibrillation that is not caused by a heart valve problem [2, 3].

Apixaban appears as a white-to-pale yellow, non-hygroscopic crystalline powder, with an aqueous solubility of 0.028 mg/mL at 24°C. Apixaban is a non-ionisable compound and its partition coefficient at 24°C is 44.7 (log Po/w = 1.65) at pH 7.4 (*n*-octanol / aqueous buffer). The molecule has no chiral centres, therefore, no stereoisomers exist [4].

There are some methods of estimation of apixaban from human plasma by LC-MS [5, 6], but there is no related substance method for apixaban by HPLC using a UV detector. Further, apixaban is not officially reported in any pharmacopeia (USP, EP, JP & IP) to date. The current HPLC method was developed and validated considering intermediates and degradation impurities.

**Fig. 2.**
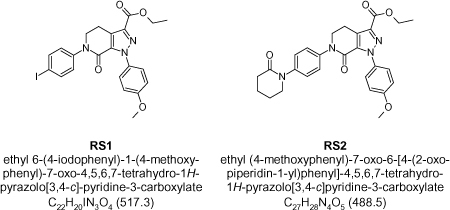
Chemical names and structures of apixaban’s process impurities

## Results and Discussion

The present study was aimed at developing a liquid chromatographic method for the separation and quantitative determination of apixaban and its related impurities.

The structures of apixaban and its known impurities (RS1, RS2) are depicted in Fig. 2. The compounds RS1 and RS2 are intermediates.

### Optimization of HPLC Conditions

The chromatographic conditions were optimized with respect to specificity, resolution, and time of analysis. The specificity of the method was established through the study of the resolution factor of the apixaban peak from the nearest resolving peak. Peaks were identified using retention times compared with those of standards and the characteristic spectra were confirmed by photodiode array detection (range 200–400 nm) and spectroscopic techniques (NMR and mass).

Several binaries were tested using different proportions of solvents, such as acetonitrile, methanol, and water. A flow rate of 1.0 mL/min was selected after preliminary tests.

**Fig. 3.**
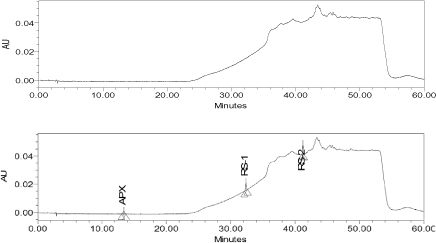
Reference chromatogram of the blank and diluted standard

Effects of pH (2.5–6) were investigated using phosphate and acetate buffers. Initial attempts were made by trying different proportions (or compositions) of acetonitrile with a buffer, but there was an interference of the blank with the RS2 peak. Columns of different compositions viz. Cosmosil, Qualisil were tried, but there was a problem with peak tailing and peak dissymmetry. Methanol-potassium dihydrogen phosphate with 0.1% TEA (and the pH of the buffer adjusted to 3.0 with diluted orthophosphoric acid (pH 4.5)) in gradient mode was found to achieve the complete separation of apixaban and known impurities within 60.0 min. In the optimized conditions, apixaban was well-separated from RS1 and RS2 as shown in Fig. 3.

### Structural Elucidation of Related Impurities and the Drug Substance

The identification of apixaban and its known impurities (RS1) and (RS2) was confirmed by ESI-MS, ^1^H NMR, and FT-IR. The spectral data on the ^1^H NMR chemical shift, FT-IR, and m/z of apixaban and the impurities are presented in tables 1, 2, and 3.

**Tab. 1.** Comparative ^1^H NMR assignments for apixaban and its impurities

For RS1 (in CDCl_3_) ^1^H NMR (CDCl_3_) δ: 7.70 (d, *J=* 8.80 Hz, 2H), 7.46 (d, *J =* 8.80 Hz, 2H), 7.08 (d, *J =* 8.80 Hz, 2H), 6.92 (d, *J=* 9.20 Hz, 2H), 4.49 (q, *J =* 6.90 Hz, 2H), 4.12 (t, *J=* 6.60 Hz, 2H), 3.81 (s, 3H), 3.35 (t, *J=* 6.60 Hz, 2H) ppm
For RS2 (in CDCl_3_) ^1^HNMR (CDCl_3_) δ: 7.49 (d, *J=* 9.20 Hz, 2H), 7.35 (d, *J=* 8.80 Hz, 2H), 7.26 (d, *J=* 8.10 Hz, 2H), 6.92 (d, *J=* 8.80 Hz, 2H), 4.49 (q, *J=* 7.30 Hz, 2H), 4.13 (t, *J=* 6.60 Hz, 2H), 3.81 (s, 3H), 3.59 (m, 2H), 3.39 (t, *J=* 6.60 Hz, 2H), 2.55 (m, 2H), 1.91 (m, 4H), 1.45 (t, *J=* 7.30 Hz, 3H) ppm
For apixaban (in CDCl_3_) ^1^HNMR (CDCl_3_) δ: 7.49 (d, *J =* 8.80 Hz, 2H), 7.37 (d, *J=* 9.10 Hz, 2H), 7.26 (d, *J=* 8.80 Hz, 2H), 6.98 (s, 1H), 6.95 (d, *J =* 9.20 Hz, 2H), 6.28 (s, 1H), 4.14 (t, *J=* 6.60 Hz, 2H), 3.81 (s, 3H), 3.61 (m, 2H), 3.39 (t, *J =* 6.60 Hz, 2H), 2.63 (t, *J=* 6.20 Hz, 2H), 1.96 (m, 4H) ppm

**Tab. 2.** Mass spectrum for apixaban and its impurities

Compound	m/z data
Apixaban	460.37 (M + 1) in +ve mode
RS1	518.29 (M + 1) in +ve mode
RS2	489.42 (M + 1) in +ve mode

**Tab. 3.** FT-IR spectral data of apixaban and its impurities

Compound	IR (KBr) absorption bands (cm^-1^)
Apixaban	1630 for -C=O stretch cyclic amide; 1595 for -C=O stretch amide lactum; 3023 for -C-H stretch aromatic
RS1	1713 for -C=O stretch ester; 1677 for -C=O stretch amide; 3013 for C-H stretch aromatic
RS2	1650 for -C=O stretch amide lactum; 1251 for -C-O bend

### Results of Forced Degradation

**Fig. 4.**
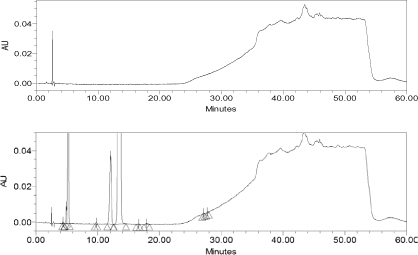
Reference chromatogram 5 N HCl + 5 N NaOH (blank) and degradation obtained after testing with 5 N HCl

**Fig. 5.**
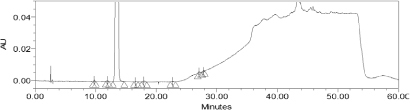
Reference chromatogram obtained after degradation testing with 5 N NaOH

**Fig. 6.**
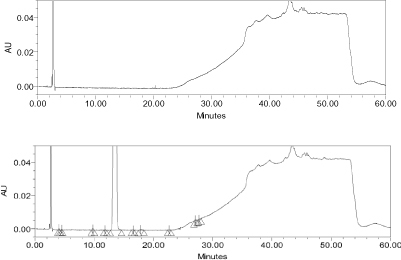
Reference chromatogram of 1% H_2_O_2_ (blank) and degradation obtained after testing with 1% H_2_O_2_

When apixaban was subjected to solution state forced degradation (acid, alkaline, and peroxide), the drug molecule degraded up to 12% under strong acidic conditions (heating at 60°C for 3.5 hours), but in alkaline conditions and peroxide it was stable (Figures 4–6). It was observed that apixaban did not degrade under UV light (photolytic) and heating at 105°C (thermal conditions) (Table 4). The unknown degradants produced in the forced degradation were well-separated (Rs > 2.0) from apixaban and its known impurities. The peak homogeneity of apixaban and its impurities was verified using a photodiode array detector by using Empower software which indicated that the peak purities of all components were passing.

**Tab. 4.** Results from stress testing of apixaban

Nature of stress	Results
UV and visible light	No degradation
Hydrolysis (5 N NaOH)	No degradation
Hydrolysis (5 N HCl)	87.4% (i.e. 12.6% degradation after 3.5 h) at 60°C
Oxidation (1% H_2_O_2_)	No degradation
Thermal (at 105°C)	No degradation

### Validation

#### Linear Range

Calibration plots were linear over the range of 0.01-0.22 μg mL^-1^. Linear regression analysis of the concentration against the peak-area ratio resulted in an average coefficient of determination *(R)* greater than 0.99. The linear regression results (Table 5) indicated that detector responses at 220 nm were linear over the concentration range studied [6].

**Tab. 5.** Linearity of apixaban and impurities

Component	Range Covered	Y-Intercept	R
RS1	LOQ to 150%	875.37	0.9997
RS2	LOQ to 150%	-216.55	0.9996
Apixaban	LOQ to 150%	-752.75	0.9997

#### Limits of Quantitation and Detection

The LOD and LOQ were determined for apixaban and each of the impurities based on the % relative standard deviation (SD) of the response.

The concentrations of LODs were found to be 0.05 μg/mL for apixaban, 0.008 μg/ mL for RS1, and 0.009 μg/mL for RS2, while the LOQs were found to be 0.15 μg/mL for apixaban, 0.024 μg/mL for RS1, and 0.026 μg/mL for RS2. It is shown in Table 6.

**Tab. 6.** Limit of detection and limit of quantification of apixaban and its impurities

Component	LOD (μg/mL)	LOQ (μg/mL)
RS1	0.008	0.024
RS2	0.009	0.026
Apixaban	0.05	0.15

#### System Suitability Test

The solution of apixaban 0.1% w/w with 0.15% w/w of known impurities was analyzed during the validation studies. It may be noted that the resolution value of more than 2.0 was achieved for all the compounds under study with no significant variation noticed in the RRTs and tailing factors of the compounds. The percentage relative standard deviation of the peak of the six replicate injections of the standard solution was observed to be no more than 5.

#### Accuracy

The recovery of each impurity was observed in the range of 93.4–100.5% (Table 7).

**Tab. 7.** Recovery of apixaban impurities at the LOQ, 50%, 100%, and 150% level

Components	LOQ level	50% level	100% level	150% level
RS1	73.8	93.8	98.3	100.4
RS2	96.3	98.4	97.8	98.3

#### Robustness

The robustness of the method was checked by doing deliberate changes in the method parameters viz. flow rate (± 20%), column temperature (± 2°C), and change in pH (± 0.2).

It was observed that the resolution between apixaban and RS1 was more than 1.5 and the %RSD of the six replicate standard injections of the impurity solutions was no more than 5. It is shown in Table 8.

**Tab. 8.** Results from the evaluation of the robustness parameter for apixaban

	Retention time of apixaban (min)	Resolution between apixaban and RS1	Number of theoretical plates	Tailing factor
Proposed method*	13.6	47.3	65887	1.12
Column temperature 28°C	14.7	37.9	51321	1.16
Column temperature 32°C	13.5	41.5	53673	1.13
Flow rate (0.8 mL/min)	16.4	36.1	60217	1.12
Flow rate (1.2 mL/min)	11.5	47.1	52471	1.15
Change in buffer pH (4.3)	14.1	30.2	33308	1.58
Change in buffer pH (4.7)	14.1	28.3	28768	1.50

#### Stability of the Analytical Solution

The % difference in the peak area of all the known and unknown impurities from the initial to relevant time interval was calculated. Based on the data, the sample solution was declared to be stable for at least 24 h at room temperature.

#### Application of the method

Three batches of APX drug substance were analysed using the proposed method. The levels of impurities relative to APX were in the range of 0.07–0.08% and total impurities were in the range of 0.39–0.43%. It is shown in Table 9.

**Tab. 9.** Results of analysis of apixaban for impurities

Batch No.	Impurity in % w/w			
	RS1	RS2	Any other individual impurity	Total impurity
#001	ND	0.01	0.07	0.39
#002	ND	0.01	0.08	0.40

ND…Not deteced

## Experimental

### Chemicals

Methanol (HPLC grade), distilled water (Rankem), potassium dihydrogen phosphate (HPLC grade, Merck), hydrochloric acid (HCl), sodium hydroxide (NaOH), hydrogen peroxide (H_2_O_2_), orthophosphoric acid (OPA), and triethylamine (TEA) were purchased from Spectrochem (Mumbai, India). The investigated samples of apixaban and intermediates (related impurities) were synthesized at Wanbury R&D Center, Mahape, New Mumbai, India.

### Chromatography

The HPLC system used in this study was the Waters HPLC equipped with Alliance 2695, separations module, 2996 photodiode array (PDA) detector with Empower software and with Alliance 2695 with a 2489 UV detector. The HPLC column used in this study was the Hypersil BDS C-18, 250 mm × 4.6 mm, 5 μm (Thermo Fisher, Thermo Technologies).

The analysis was carried out on the Hypersil BDS C18 (250 mm × 4.6 mm, 5 μm) column thermostated at 30°C. Solvent A was potassium dihydrogen orthophosphate buffer (0.01 M KH_2_PO4 + 1 mL of triethylamine (TEA) pH-adjusted to 4.5 with diluted orthophosphoric acid) and solvent B was methanol. Solvent A was filtered through a 0.45 μm membrane filter and degassed prior to pumping into the system along with solvent B. The mobile phase flow rate was 1.0 mL/min. The HPLC gradient program was time (min)/%B (v/v): 0/45, 18/45, 40/80, 50/80, 51/45, and 60/45. The injection volume was 10 μl. The chromatograms were recorded at 220 nm using a UV detector.

### Analytical Procedure

Apixaban reference standard at 1.0 mg/mL and subsequent concentrations were prepared in diluent (water: acetonitrile), respectively. Solutions of all the impurities (0.15% w/w) were prepared by dissolving known amounts of the compounds initially in diluent and diluted up to the mark with the same diluent. These solutions were prepared freshly and diluted further quantitatively to study the validation attributes. The specification limit considered for the validation studies was 0.15% for the known impurity. Known and unknown impurities were determined against the mean areas obtained from six replicate injections of the standard solution and relevant impurity standard solution.

### Method Validation

Validation of the chromatographic method was carried out with regards to linearity, sensitivity (limit of detection and limit of quantification), precision, accuracy, and robustness.
